# Boar seminal plasma: current insights on its potential role for assisted reproductive technologies in swine

**DOI:** 10.1590/1984-3143-AR2020-0022

**Published:** 2020-07-21

**Authors:** Inmaculada Parrilla, Emilio Arsenio Martinez, Maria Antonia Gil, Cristina Cuello, Jordi Roca, Heriberto Rodriguez-Martinez, Cristina Alicia Martinez

**Affiliations:** 1 Departmento de Medicina y Cirugía Animal, Facultad de Veterinaria, Campus de de Excelencia International “Campus Mare Nostrum”, Universidad de Murcia, Murcia, Spain; 2 Instituto Murciano de Investigación Biosanitaria, Campus de Ciencias de la Salud, Murcia, Spain; 3 Department of Biomedical & Clinical Sciences, BKH/Obstetrics & Gynaecology, Faculty of Medicine and Health Sciences, Linköping University, Linköping, Sweden

**Keywords:** protein, cytokine, sperm, embryo, pig

## Abstract

Seminal plasma (SP) supports not only sperm function but also the ability of spermatozoa to withstand biotechnological procedures as artificial insemination, freezing or sex sorting. Moreover, evidence has been provided that SP contains identifiable molecules which can act as fertility biomarkers, and even improve the output of assisted reproductive technologies by acting as modulators of endometrial and embryonic changes of gene expression, thus affecting embryo development and fertility beyond the sperm horizon. In this overview, we discuss current knowledge of the composition of SP, mainly proteins and cytokines, and their influence on semen basic procedures, such as liquid storage or cryopreservation. The role of SP as modulator of endometrial and embryonic molecular changes that lead to successful pregnancy will also be discussed.

## Introduction

The OECD-FAO predicts a 15% increase in global meat production, including pig meat, for 2027 (FAO, 2018). Such increase largely depends of a successful reproductive management of the herds by correctly implementing the reproductive biotechnologies available today (Choudhary et al., 2016), such as artificial insemination (AI) or embryo transfer (ET) to ensure a sustained production of large litters. Therefore, it is mandatory to develop new strategies that help to reach such outputs, alongside higher economical and, potentially, environmental benefits by keeping the number of breeders at optimal levels, e.g. decreasing the number of low-effective breeders (Bromfield, 2016).

The early detection of boars with compromised fertility is then a logical priority for AI programs (Roca et al., 2015). However, it is generally accepted that conventional semen evaluation methods offer only a rough assessment of the real fertility potential of a boar (Dyck et al., 2011). In an attempt to overcome this drawback, special attention has been given to the study of the composition of seminal plasma (SP) as a potential source of biomarkers that could help to identify sub-fertile AI-boars that escape conventional semen screening yet providing sub-fertile semen (Pérez-Patiño et al., 2019). Sperm physiology and fertilizing ability are highly influenced by SP composition, which promotes sperm function and survival (Schjenken and Robertson, 2014). Moreover, the use of boar SP as an additive to optimize the quality of functional characteristics of biotechnologically treated spermatozoa has also been described (Parrilla et al., 2009; Caballero et al., 2012). Very recent reports of studies performed at a molecular level indicate that the SP effectively modulates the uterine environment in gilts and sows at different stages of the estrous cycle (pre-, peri-, post-ovulatory, and early pregnancy stages; Waberski et al., 2018; Alvarez-Rodriguez et al., 2019; Martinez et al., 2019c), potentially establishing best conditions for embryonic development and implantation. These findings are extremely relevant, especially in the field of reproductive technologies in pigs, since their implementation could result in important improvements in reproductive efficiency when using AI or ET largely impacting the production sector.

The present review summarizes current knowledge about boar SP, focusing mainly on those aspects related with the preservation of sperm function, as well as a source of potential biomarkers of boar fertility. In addition, the role of SP among the signaling mechanisms to help establish a successful pregnancy will also be discussed.

## Seminal plasma characteristics and composition

SP is a complex fluid in which spermatozoa are sequentially suspended during the ejaculation process and it is composed of a mixture of secretions from the testis, epididymis, but mainly from the sexual accessory glands (Mann and Lutwak-Mann, 1981). In boar, the SP represents the major portion of the ejaculate volume (approximately 95%) being physiologically emitted in fractions, which can be collected and analyzed separately; these fractions are different in terms of origin and composition (Rodriguez-Martinez et al., 2009).

The most relevant components of boar SP are listed in Table 1. Below, we will discuss the influence of some of these SP constituents on sperm function. The SP proteins and cytokines will be reviewed in separate sections due to their major role in regulating sperm function and fertility outcomes.

**Table 1:** Most relevant components of boar seminal plasma.

**Component**	**Source**	**Suggested functions**	**Reference**
**Ions**			
Na	Testes; Cauda Epididymis; Vesicular Glands; Bulbouretral Glands	Osmotic balance; sperm motility; sperm morphology; sperm metabolism	López-Rodriguez et al. (2013)
Cl			
Ca	Testes; Cauda Epididymis; Vesicular Glands	Acrosome reaction; sperm motility	López-Rodriguez et al. (2013)
K	Testes; Cauda Epididymis; Vesicular Glands; Bulbouretral Glands	Sperm motility	Juyena and Stelletta (2012), Johnson et al. (2000)
Bicarbonate	Prostate; Vesicular Glands	Sperm motility; induction of plasma membrane destabilization	Rodriguez-Martinez et al. (2009)
Mg	Cauda Epididymis; Vesicular Glands; Bulbouretral Glands	Enzymatic systems; sperm motility integrity of sperm membrane	Juyena and Stelletta (2012), López-Rodriguez et al. (2013)
Se	Epididymis; Prostate; Vesicular Glands	Sperm motility; sperm morphology; sperm viability and integrity of DNA; structural component of glutathione peroxidase	López-Rodriguez et al. (2013), Pipan et al. (2017), Barranco et al. (2016), Qazi et al. (2019)
Zn	Vesicular Glands	Mitochondrial function; protection against oxidative stress; antibacterial activity of SP; stabilization of DNA nucleoproteins; control of energy for sperm motility; *in vivo* fertility	Boursnell et al. (1972), Guthrie et al. (2008), López-Rodriguez et al. (2013)
**Enzymes**			
ALP	Epididymis	spermatozoa quiescence Modulation of fertilizing capability acquisition	López-Rodriguez et al. (2013), Bucci et al. (2014)
AST	Epididymis	Indicator of sperm cell damage	López-Rodriguez et al. (2013), Ciereszko et al. (1992)
	Vesicular Glands		
AP	Epididymis	Sperm plasma membrane integrity	Wysocki and Strzezek (2000)
	Vesicular Glands	Control of sperm metabolism	
GGT		Protective effect of sperm against oxidative stress	López-Rodriguez et al. (2013)
LDH	Testes; Epididymis	Necessary for energy metabolism of sperm	Sopkova et al. (2015), Mann and Lutwak-Mann (1981)
(isoenzyme LDH 4)		Maintenance of sperm motility	
		Involved in sperm capacitation	
SOD	Epididymis; Vesicular Glands; Prostate	Sperm protection against reactive oxygen species	Roca et al. (2005)
PON-1	Testes; Epididymis	Prevent oxidation of low-density lipoprotein cholesterol	Barranco et al. (2015a)
GPX-5	Testis; Epididymis	Neutralizing H_2_O_2_	Barranco et al. (2016)
	Vesicular Glands; Prostate; Bulbourethral Glands	Sperm protection against reactive oxygen species	
**Energy Substrates**			
Glucose, fructose, sorbitol	Vesicular Glands	Energy source and modulators of sperm function	Rodríguez-Gil (2013)
**Other relevant components**			
Citric Acid	Cauda Epididymis; Vesicular Glands	pH control in boar semen; Zn, Mg and Ca chelator	Setchell and Brooks (1988), Kamp and Lauterwein (1995)
Inositol	Vesicular Glands	Maintenance of osmotic balance	Mann and Lutwak-Mann (1981), Setchell and Brooks, 1988
Phosphate	Testes; Cauda Epididymis; Vesicular glands; Bulbouethral Glands	Sperm motility	Setchell and Brooks (1988), López-Rodriguez et al. (2013)
Glycero-phosphocholine	Cauda Epididymis; Vesicular Glands	Reserve of substrate	Mann and Lutwak-Mann (1981), Cooper (1986)
		Reduce sperm motility in-vitro	
		Regulation of osmotic pressure	
Ergothioneine	Cauda Epididymis; Vesicular Glands	Prevention of lipid peroxidation	Mann and Leone (1953), Nikodemus et al. (2011)
		Maintenance of intracellular SH-groups in a physiologically active condition	
Hypotaurine	Cauda Epididymis	Osmoregulation and reducing agent	Johnson et al. (1972)

### Ions

Macroelements such as sodium, calcium, potassium, magnesium and chlorine greatly influence sperm functions (Hamamah and Gatti, 1998). In boar, sodium and chlorine are the most abundant ions and, among other functions, they influence metabolism and hence sperm motility and membrane stability, which can modify the morphology of spermatozoa (López-Rodriguez et al., 2013). Potassium, a metabolic inhibitor, decreases sperm metabolism and hence lowers sperm motility (Juyena and Stelletta, 2012; Johnson et al., 2000). Calcium, of utmost relevance for sperm motility variations, is pivotal when triggering the acrosome reaction (Juyena and Stelletta, 2012). Magnesium is involved in almost all enzymatic reactions, and thus related to sperm motility and sperm membrane preservation (Juyena and Stelletta, 2012; López-Rodriguez et al., 2013). Other ions present in boar SP, such as copper, selenium and zinc, also influence sperm quality (Pipan et al., 2017). Selenium is a component of glutathione peroxidase, an enzyme with antioxidant properties whose presence in the SP has been associated with sperm survival and *in vivo* fertility (Barranco et al., 2016). Moreover, recent studies have shown that selenium in the SP is related to sperm motility, morphology, and viability and the integrity of DNA (Pipan et al., 2017). Zinc is essential for chromatin intactness, preserves mitochondrial function and acts as a protective agent against oxidative stress (Guthrie et al., 2008) and contributes to the antibacterial activity of SP (Juyena and Stelletta, 2012). Finally, higher levels of other ions, including iron and copper, have been correlated with a higher number of functional sperm after storage (Pipan et al., 2017).

### Enzymes

There are a wide variety of enzymes in the SP that have different functions. Very recently, it has been observed that more than 3% of the pig SP proteome are enzymes (Roca et al., 2020). Among them, there are several with antioxidant properties, whose main action is to reduce lipid peroxidation to protect spermatozoa from excessive levels of reactive oxygen species (ROS), particularly relevant in pigs due to the high sensitivity of their spermatozoa to oxidative stress (Radomil et al., 2011; Li et al., 2018). Superoxide dismutase (SOD), catalase (CAT), glutathione peroxidase (GPx), glutathione reductase, glutathione S-transferase, phospholipid hydroperoxide glutathione peroxidase, gamma-glutamyl transferase (GGT) and paraoxonase type 1 (PON-1) are among the antioxidant enzymes present in boar SP (López-Rodriguez et al., 2013; Koziorowska-Gilun et al., 2011; Barranco et al., 2015a, c, 2016).

Many of these enzymes have positive effects on the function of frozen-thawed (SOD; Roca et al., 2005) and refrigerated (PON-1 and GPx5; Barranco et al., 2016) spermatozoa, on the sperm concentration in the ejaculate and progressive motility (GGT; López-Rodriguez et al., 2013). Moreover, SOD, PON-1 and GPx-5 have been positively associated with the sperm response to cryopreservation, as they minimize oxidative stress associated with this procedure (Li et al., 2018).

Other enzymes present in boar SP are those related to preserving the stability of the sperm plasma membrane and sperm metabolic function. For instance, lactate dehydrogenase (LDH) and especially its isoenzyme LDH-C4 are important indicators of fertility, displaying higher concentrations among normospermic boars with high sperm motility (Sopkova et al., 2015). Aspartate amino transferase (AAT) activity in SP indicates sperm damage (Forejtek and Navratil, 1984) being negatively correlated to normal mophology, intact acrosomes and normal fertility (Juyena and Stelletta, 2012; López-Rodriguez et al., 2013). High activity of acid phosphatase has been correlated with sperm concentration, motility, and integrity of the acrosome membrane (Wysocki and Strzezek, 2000), while alkaline phosphatase activity might play a role in preventing premature capacitation and, therefore, preserving fertilizing ability (Bucci et al., 2014).

### Energy substrates

To maintain their functionality and especially to ensure adequate motility, boar spermatozoa require energy that is usually obtained from exogenous substrates present in SP. Monosaccharides, such as glucose and fructose, and polyols, such as sorbitol, are the main energy sources for sperm present in boar SP, and glycolysis the main pathway of glucose utilization, producing pyruvate/lactate (Mann and Lutwak-Mann, 1981; Setchell and Brooks, 1988; Marin et al., 2003). However, boar spermatozoa can, in the absence of monosaccharides, also use other substrates, such as glycerol, lactate, pyruvate and citrate (Rodríguez-Gil, 2013). It is worth noting that knowledge of energy regulation sperm mechanisms is fundamental to design strategies for adequate handling and storage conditions for best preservation of sperm quality.

There are many other constituents of SP that modulate sperm functions, and some details about them have been included in Table 1.

## Seminal plasma and semen technologies

The addition of SP to boar sperm subjected to different biotechnological treatments, such as sperm sex sorting or cryopreservation, has been proposed as a strategy to reduce negative effects (Maxwell and Johnson, 1999; Parrilla et al., 2009). However, contradictory results have been described (Novak et al., 2010; Yeste et al., 2017), probably mainly attributed to variations in the protein composition of SP (Druart et al., 2013; Pérez-Patiño et al., 2016).

### Seminal plasma proteins

Proteins are one of the most important components of boar SP, with concentrations ranging from 30 to 60 g/L (Rodriguez-Martinez et al., 2009). Many SP proteins bind to the surface of spermatozoa to modulate their functional capacity (Caballero et al., 2012; Parrilla et al., 2019). These proteins also protect spermatozoa during their transit through the sow genital tract, contributing to the regulation of the temporal kinetics of ovulation and subsequent corpus luteum development and facilitating, in combination with other SP components, early pregnancy success (Waberski et al., 1997; Troedsson et al., 2005; Bromfield, 2016). A direct relationship between several SP proteins and fertility has been demonstrated in several species (Mogielnicka-Brzozowska and Kordan, 2011).

**Figure 1:**
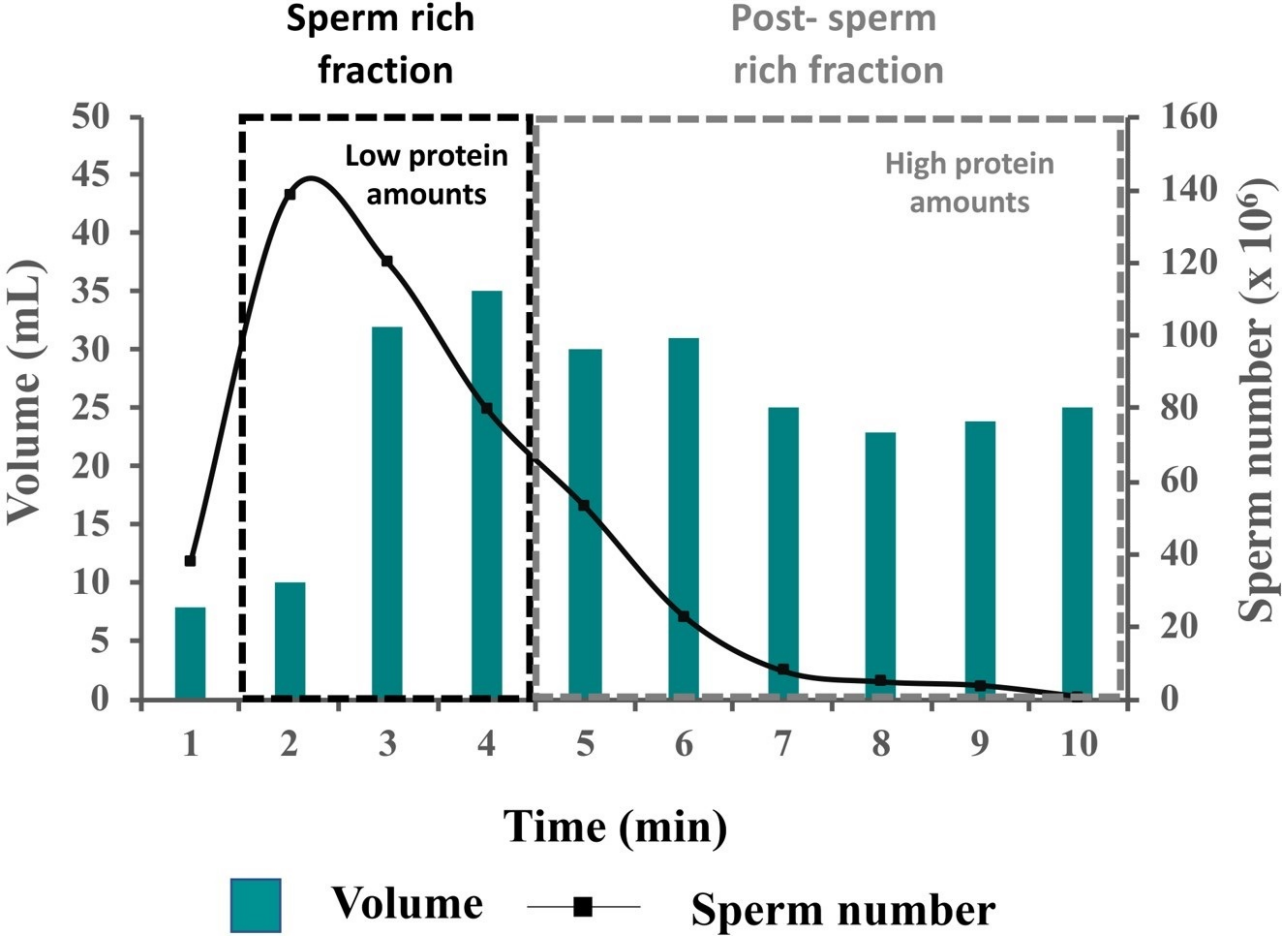
Boar ejaculate: Sperm concentration, ejaculate volume and relative amount of proteins in the consecutive portions of boar ejaculate namely, sperm rich fraction and post- sperm rich fraction. Modified from: Rodriguez-Martinez et al. (2009, 2011).

In boar, most SP proteins are spermadhesins (75-90% of total SP protein content; Rodriguez-Martinez et al., 2009), a highly multifunctional family of glycoproteins classified according their ability to bind (AQN-1, AQN-3 and AWN) or not (PSP-I and PSP-II), heparin. Within a boar ejaculate, the relative protein concentrations are low in the pre-sperm fraction and the first portion of the sperm rich fraction (SRF) and increase in latter fractions of the ejaculate (Rodriguez-Martinez et al., 2011) (Figure 1).

*In vivo*, several spermadhesins have been associated with sperm fertilization capability (Caballero et al., 2012). Moreover, numerous studies have focused on determining their potential use as biomarkers of male fertility and as additives for the improvement of biotechnologically treated spermatozoa (Dyck et al., 2011; Caballero et al., 2012; Vilagran et al., 2014). These studies have provided valuable information regarding their effects on sperm functional parameters and, more importantly, their potential relationship with boar sperm fertilization capability *in vitro* and *in vivo* (Garcia et al., 2007; Novak et al., 2010; Dyck et al., 2011; Caballero et al., 2012). However, although these findings were promising, they were focused on the identification of a single protein, while effective fertilization is the result of multiple interactions between different proteins. Therefore, more research is required to ensure the usefulness of these and other proteins as fertility markers.

To identify such proteins useable as fertility biomarkers, we performed proteomic analysis of the entire ejaculate and its fractions (first 10 mL of SRF, the rest of SRF and post-SRF) (Pérez-Patiño et al., 2016). We identified a total of 536 proteins in the entire ejaculate, 374 of which represented in the *Sus scrofa* taxonomy. Despite the high number of proteins identified, just 20 were directly related to reproductive processes, a striking finding when considering that the main effects of SP proteins are on sperm function and attainment of pregnancy (Caballero et al., 2012; Bromfield, 2016). Most likely, this finding was due to the incomplete association of the proteins identified with specific terms in the Gene Ontology knowledge base rather than to an actual deficiency among proteins related to reproductive functions (Pérez-Patiño et al., 2016). Despite this, the bioinformatic analysis showed that many of other identified proteins were related to important functions directly involved in reproductive processes, such as ion-and calcium-binding properties, glycosylation, immune responses and antioxidant activity, among others. In that study, we also showed quantitative rather than qualitative differences in protein SP-composition from the different ejaculate portions analyzed. There were no differences in protein levels between the first 10 mL of SRF and the rest of the SRF. In contrast, we found 34 proteins that were differentially expressed between the SRF and post-SRF. Sixteen of these proteins were represented in the *Sus Scrofa* taxonomy, and eight of them were overexpressed in SRF compared to their levels in the post-SRF. Some of the overexpressed proteins have been previously related to sperm capacitation, the acrosome reaction, zona pellucida binding, membrane stability and permeability (HEXB, GP2, ARSA, GLB1L3; Pérez-Patiño et al., 2016). Proteins overexpressed in the post-SRF were related to sperm maturation, sperm motility and bactericide activity. The remaining 18 differentially expressed proteins, which were not present in the *Sus Scrofa* taxonomy, were found to be involved in spermatogenesis, sperm maturation or mature sperm functionality in other species of mammals (Pérez-Patiño et al., 2016). These results seem to indicate that quantitative variations in SP-proteins are most likely responsible for the different effects of SP from different fractions on boar spermatozoa (Garcia et al., 2007; Saravia et al., 2009; Novak et al., 2010; Alkmin et al., 2014).

In a subsequent study, Pérez-Patiño, et al. (2019) attempted to increase the number of less abundant SP-proteins, very relevant for biological processes, that could be detected by including a prefractionation step by solid phase extraction (Brewis and Gadella, 2010). A total of 872 proteins were identified, 336 more than in our previous study (Pérez-Patiño et al., 2016). Of these, 37 proteins related to reproductive functions. Notably, among them were the low-molecular weight and highly abundant spermadhesins (PSP-I, PSP-II, AWN, AQN-1, and AQN-3), which, as mentioned above, exert important effects on boar sperm functions (Caballero et al., 2012), ensuring the maintenance of the proper immune environment in the uterus for embryonic development (Rodriguez-Martinez et al., 2011). More importantly, we detected proteins differentially expressed in SP samples from boars with different farrowing rates and litter sizes (data recorded from 10,526 sows; Pérez-Patiño et al., 2019). Eleven proteins were differentially expressed in boars with high- and low- farrowing rates (8 overexpressed and 3 underexpressed in boars with high farrowing rates). Among the overexpressed proteins, 4 proteins (furin, UBA1, SPAM-1, and AKR1B1) showed a direct implication in male reproductive success, such as sperm maturation, capacitation, motility, and fertilizing ability (Pérez-Patiño et al., 2019). We speculate that a higher expression of these proteins would contribute to successful fertilization, as reflected by the higher farrowing rates obtained when these boars were used for AI. Regarding the differentially expressed SP proteins in boars with larger and smaller litter sizes, we identified 4 other proteins; two of them (DSC-1 and CAT) overexpressed in those boars with larger litter sizes. The DSC-1 is involved in the proper functioning of spermatogenesis, while CAT is a well-known antioxidant enzyme, protecting against ROS (Awda et al., 2009). The PN-1 was one of the underexpressed SP proteins, which has been related to seminal vesicles dysfunction in humans when present in excess and with infertility when lacking (Murer et al., 2001). The other underexpressed protein was THBS1, whose presence in SP during AI alters maternal-conceptus communication in early pregnancy stages (Edwards et al., 2011). The low expression of these SP-proteins in boars with larger litter sizes suggests they could be used as potential fertility biomarkers.

### Seminal plasma cytokines

Cytokines are a family of proteins of low molecular weight (between 5 and 20 kDa) mainly known for being involved in the immune response as regulatory factors (Jiang et al., 2016; Syriou et al., 2018). The role of cytokines in different reproductive events, including ovarian and testis functionality, embryonic development, endometrial immune responses and proper placental function and parturition, has been described by several authors (Robertson and Moldenhauer, 2014). In humans, cytokines are directly related to semen quality and functionality and play an important role in fertility regulation (Fraczek and Kurpisz, 2015). However, the potential influence of these cytokines on boar fertility profile is far from clear. To increase the understanding of SP cytokines in boars, we used a multiplex assay approach to identify and quantify different cytokines in SP from the SRF and post-SRF (Barranco et al., 2015b). Our results demonstrated that the SP of all analyzed boars contained a variety of measurable cytokines with pro- and anti-inflammatory activity. This fact suggests that, similar to that in humans and mice, that pig-SP could modulate uterine immune mechanisms to facilitate the transition from a primary anti-inflammatory response after AI to a more immunotolerant environment prior to embryo implantation (Robertson, 2005; Bromfield, 2014). More interestingly, our study found that cytokine concentrations varied among boars and ejaculated fractions, as they were more abundant in the post-SRF. Several authors have described the different tolerances of spermatozoa retrieved from the post-SRF to biotechnological procedures such as liquid storage or cryopreservation (Saravia et al., 2009; Alkmin et al., 2014). The possibility that SP cytokines, by interacting with spermatozoa, are partly responsible for these differences deserves to be thoroughly studied. In this line, we have shown how specific boar SP cytokines could modulate sperm changes at different levels during preservation (Barranco et al., 2019; Table 2).

**Table 2:** Predictive value of seminal plasma cytokines for sperm quality and functionality parameters in liquid-stored and cryopreserved boar semen samples.

**Seminal plasma cytokine**	**Sperm sample**	**Total motility**	**Progressive motility**	**Viability**	**H_2_O_2_ generation**	**Total O_2_ generation**	**Lipid peroxidation** ^*^
TGF-β1	Liquid						
	Cryopreserved	**-**	**-**				**+**
TGF-β2	Liquid	**-**	**-**				
	Cryopreserved						
TGF-β1	Liquid	**+**	**+**		**+**		
	Cryopreserved						
GM-CSF	Liquid						
	Cryopreserved				**+**		
IFN-γ	Liquid				**+**		
	Cryopreserved	**+**	**+**	**+**	**+**	**-**	**-**
IL-1α	Liquid						
	Cryopreserved			**+**	**-**		
IL-1Ra	Liquid	**-**	**-**				
	Cryopreserved	**-**	**-**	**-**			**-**
IL-2	Liquid						
	Cryopreserved	**-**					
IL-4	Liquid	**-**	**-**				
	Cryopreserved		**-**	**-**	**-**		
IL-6	Liquid						
	Cryopreserved					**+**	
IL-8	Liquid	**+**	**+**				
	Cryopreserved	**-**	**-**	**-**		**+**	**+**
IL-10	Liquid	**+**					
	Cryopreserved					**+**	
IL-12	Liquid		**-**		**-**	**-**	
	Cryopreserved						
IL-18	Liquid	**+**	**+**				
	Cryopreserved					**-**	

+ /-: Positive and negative relationship.

* Viable sperm showing lipid peroxidation. Modified from Barranco et al. (2019). TGF: Transforming Growth Factor; GM-CSF: Granulocyte-Macrophage Colony Stimulation Factor; IFN: Interferon; IL: Interleukyn.

## Seminal plasma and embryo technologies

Currently, it is widely recognized that SP not only plays a key role as a nutrient and vehicle for spermatozoa but also exerts important functions on the tissues of the female genital tract, impacting subsequent events such as fertilization, implantation and pregnancy (Robertson, 2005, 2007). Numerous studies in human, rodents and domestic species indicate that the SP contains specific constituents with potential to induce modifications at the molecular, biochemical and cellular levels in the female genital tract (Robertson, 2005, 2010; Rodriguez-Martinez et al., 2011). Thus, the infusion of SP prior to AI alters the expression of many genes related to maternal immunity in the reproductive tract of peri-ovulating sows (Waberski et al., 2018; Alvarez-Rodriguez et al., 2019). The SP promotes the release of factors related to the development of preimplantation embryos during attachment (Schjenken and Robertson, 2014). However, the most substantial information regarding the effects of SP on embryonic developmental competency is derived from studies in rodents. In these species, the infusion of SP during estrus supports not only embryo development but also implantation (Pang et al., 1979; Queen et al., 1981). In the absence of SP, the rates of fertilization and preimplantation embryo development are reduced, and postimplantation pregnancy losses are increased (Peitz and Olds Clarke, 1986; O et al., 1988). Furthermore, in these species, surrogate females are usually treated with SP infusions during estrus in embryo transfer (ET) programs to increase embryo survival and implantation rates post-ET (Watson et al., 1983; Carp et al., 1984; Bromfield et al., 2004). Overall, these findings indicate that the effects of very early signaling of the infusions of SP during estrus remain influential over time and affect later processes related to preimplantation embryo development and implantation, at least in rodents. Despite this evidence, studies of the molecular changes in the preimplantation porcine endometrium and embryos in response to SP, which would be of enormous importance for porcine ET technology, have been limited.

### Seminal plasma and the transcriptional pattern of the preimplantation endometrium

Infusion of SP at the onset of estrus interacts with the endometrium and induces the modification of certain cytokines, such as granulocyte macrophage colony-stimulating factor (O’Leary et al., 2004), which is a promoter of the development and viability of mammalian preimplantation embryos (Sjoblom et al., 2002). Furthermore, these authors indicated that endometrial cytokine changes induced by SP infusions lasted for at least the first 9 days of pregnancy and were accompanied by an increase in embryo viability and changes in the kinetics of embryos, delaying their development.

We recently examined the effects of SP on the development and viability of porcine preimplantation embryos and the changes of the global transcriptome of the endometrium (Martinez et al., 2019c). In this study, post-weaning estrus sows received intrauterine infusions of SP or Beltsville Thawing Solution (BTS; Pursel and Johnson, 1975) 30 minutes before each insemination. Embryos and endometrium samples were removed during laparotomy 6 days after the infusions to morphologically evaluate the embryos and analyze the endometrial transcriptome, at Day 6 of the cycle when embryo collection and transfer are usually performed in pig ET programs. The endometrial morphology was affected by the infusion of SP, showing accentuated inflammatory changes compared to endometria from the BTS group. The changes included congestion, leukocyte margination, edema, hemorrhages and infiltrates of immune cells in the mucosal connective tissue and the uterine glands. These results support previous findings in humans and pigs, indicating that the effects of SP infusions during estrus can be observed throughout the preimplantation period in pigs (Aumüller and Riva, 1992; Maegawa et al., 2002; O’Leary et al., 2004).

On the other hand, all studies of the endometrial transcriptome during the porcine peri-implantation period have revealed alterations in the expression of genes associated with the maternal immune response (Samborski et al., 2013; Kiewisz et al., 2014; Lin et al., 2015). However, endometrial receptivity during the preimplantation period, which is greatly influenced by alterations in cytokines and other compounds secreted into the uterine fluid (Morris and Diskin, 2008; Bazer and Johnson, 2014), is also critical for the development of embryos and the appropriate progression of pregnancy. Surprisingly, we identified more than 1,600 expressed transcripts with differential abundance in the endometria of the SP and BTS groups. The endometria from SP sows showed an overrepresentation of genes associated with immune pathways, including genes such as *DLG1, FAS, LGALS1, STAT5A* and *IRF1*. Regulatory T (Treg) cells, which are a subset of CD4+ T cells, are efficient immune suppressors that play important roles in the cell-mediated immune response (Rudensky, 2011) and, therefore, are key to preventing immune rejection of the developing hemi-allogeneic embryo/fetus (Shevach, 2002; Aluvihare et al., 2004). Treg cells play a pivotal role in the progression of pregnancy by suppressing the proliferation of T and B cells (Shevach, 2002; Lim et al., 2006), inhibiting the maturation and activation of dendritic cells (DCs) and macrophages (Taams and Akbar, 2005) and preventing the cytotoxicity of natural killer cells (Ghiringhelli et al., 2005). In this regard, SP-treated sows showed evidence of the overactivation of the transforming growth factor-ß (TGF-ß) signaling pathway, which is a pathway that supports the proliferation of Treg cells by regulating DC function (Ghiringhelli et al., 2005). Altogether, these findings indicated that SP infusions prior to AI increase the development of Treg cells and control the immune response of the female in response to the presence of hemi-allogeneic embryos as early as Day 6 of pregnancy. The implications of these findings could help us reduce the immune response of subrogate females to allogeneic transferred embryos, thus decreasing the high embryonic death characteristic of current ET.

It is known that the development of embryos during the preimplantation period is severely influenced by cytokines present in uterine fluids, which either promote or limit embryonic development (Hardy and Spanos, 2002; Sjoblom et al., 2005; O’Neill, 2008). In our study, numerous genes associated with the cytokine-cytokine receptor signaling pathway were either over- or under-expressed in uterine samples exposed to SP. For example, the CD27 and CD70 genes were downregulated. The proteins encoded by these genes are costimulators of an embryotoxic cytokine (TNF-α) that increases apoptosis and inhibits embryonic development and implantation (Chaouat et al., 1990).

The development of embryos from the zygote stage to the implantation stage is controlled by many hormones, which adjust the maternal physiology to support pregnancy (Waclawik et al., 2017). Pre-AI SP infusions induce hormonal changes in the uterine environment via the overexpression of genes related to steroid and estrogen signaling.

SP-infusions also noticeably altered other pathways implicated in embryonic development and implantation. For instance, we found upregulation of the PI3K/AKT, MAPK/ERK and Wnt signaling pathways, which seem to play a fundamental role in regulating not only cell functions, including proliferation, differentiation, mitogenesis, and cell survival (Songyang et al., 1997; Nayeem et al., 2016), but also sperm functions, such as the capacitation process (Almog and Naor, 2010), and embryonic development and cytoskeletal remodeling of preimplantation trophoblast cells (Qiu et al., 2004; Bazer et al., 2010). Moreover, deficiencies in MAPK/ERK proteins result in embryonic loss by altering the trophoblast proliferation process (Saba-El-Leil et al., 2003; Jeong et al., 2013).

SP infusions induced the overexpression of many other genes of interest in the endometrium, such as *HOXB4, GRHL2, RAB14, MGAT1* and *ACVR2A*. All these genes have been associated with normal early embryonic development (Scott and Carroll, 1987; Blitek et al., 2011; Grasa et al., 2012; Petrof et al., 2014; Yong et al., 2018; Ming et al., 2018). Although the adhesion of embryos to the endometrium occurs on Days 12-16 of pregnancy, SP infusions at estrus altered, on Day 6 of pregnancy, genes that are particularly involved in cell adhesion pathways that can be responsible for increasing implantations success. Altogether, these results show that SP infusions during estrus affect the transcriptional expression profile of porcine endometrium during early pregnancy.

### Seminal plasma and preimplantation embryos

SP-infusions during estrus result in a higher percentage of advanced stage embryos as early as 6 days post-AI compared to that in controls (BTS infusions) without affecting neither fertilization rate nor embryo viability (Martinez et al., 2019c). These changes in embryonic development might be associated with variations in ovulation time in response to SP treatment. The SP alters the endocrine-immune-cytokine system in preovulatory follicles (Einer-Jensen and Hunter, 2005; O’Leary et al., 2006) by decreasing the LH peak ovulation interval and, therefore, hastening the time of ovulation (Schuberth et al., 2008). Accordingly, embryos collected from sows treated with SP should have reached a more advanced developmental stage than those collected from non-treated sows. However, SP could also directly affect embryo development because, as discussed above, the effects of SP extend beyond the time immediately after exposure. Our findings, which indicated that SP did not affect embryo viability but advanced the developmental stage of the embryos, are in contrast with previous results reported by O’Leary and coworkers (O’Leary et al., 2004), who observed that SP increased embryo viability and delayed embryonic development at Day 9 of pregnancy. The discrepancies between these studies could be attributed to differences in the methodology used for evaluating embryonic development. While we used the classical morphological evaluation method to determine the stage of the embryos, the authors of the other study evaluated the developmental stage by measuring the diameter of hatched Day 9 blastocysts, when a number of them collapsed or presented irregular morphology (Sun et al., 2015). In addition, similar to previous studies on ET (Martinez et al., 2014, 2015, 2019a, b, d), we had a high embryo viability rate on day 6 of pregnancy (>90%), which complicated the detection of significant differences between the SP and control groups.

Interestingly, recent studies from our laboratory indicate that SP infusions during estrus also modify the gene expression of Day 6 blastocysts. Transcriptome analysis of these embryos revealed 210 annotated transcripts that were differentially expressed in blastocysts derived from SP sows relative to those found in blastocysts derived from BTS sows (93 upregulated and 117 downregulated). Most of these genes were associated with biological, cellular, metabolic and developmental processes. When we analyzed the differentially expressed genes to identify the significant KEGG pathways, a total of 3 and 13 pathways were enriched in the down- and upregulated gene lists, respectively. Three pathways involved in mineral absorption, regulation of lipolysis in adipocytes and p53 signaling were enriched in the downregulated gene list and included genes, such as *MT-2B, PTGS1, ADORA1, CDK2* and *SERPINE1*, with no evident association with embryonic development or implantation. The pathways enriched among the upregulated genes included pathways related to signal transduction (apelin signaling, FoxO signaling and mTOR signaling), cellular processes (cell cycle, p53 signaling, cellular senescence, adherents junction and signaling pathways regulating pluripotency of stem cells), and the endocrine system (insulin signaling, progesterone-mediated oocyte maturation and relaxin signaling). These pathways contain genes with potential roles in embryonic development, implantation, or progression of pregnancy, such as *MAPK1, SMAD2, CDK1, ApoA-I, PRKAA1* and *RICTOR*. Our results demonstrate that SP infusions upregulated the expression of these genes, which may favor embryonic developmental capability.

The *MAPK1* gene encodes a member of the MAP kinase family that is involved in several cellular processes, such as proliferation, differentiation, transcription regulation and development. Studies in several species have shown that deficits in MAPK proteins cause early embryonic mortality due to the lack of signal transduction for proliferation and invasion of trophoblasts (Saba-El-Leil et al., 2003; Jeong et al., 2013). It has been shown in mice that *MAPK1* is essential not only for embryonic development but also for placental development (Hatano et al., 2003) and development of the mesoderm (Yao et al, 2003).

The protein encoded by the SMAD2 gene mediates the TGF-ß signaling pathway, which is an essential pathway controlling the initial developmental steps, such as epiblast development and patterning of the three germ layers (Liu et al., 2016). In addition, as mentioned above, the TGF-ß superfamily supports the proliferation of Treg cells, which are crucial for preventing immune rejection and thus tolerating the fetal allograft.

Another upregulated gene in SP blastocysts was *CDK1*, a gene indispensable not only for the mitotic cell cycle (Santamaría et al., 2007; Gavet and Pines, 2010) but also for the resumption of meiosis in oocytes (Adhikari et al., 2012), which supports the conclusion that *CDK1* may contribute to embryo viability.

Other interesting genes were *ApoA1, PRKAA1* and *RICTOR*. The *ApoA1* gene encodes apolipoprotein A-I, a principal constituent of high-density lipoprotein, which has been shown to be upregulated in the murine endometrium during implantation (Gou et al., 2015). Moreover, it has been suggested that *ApoA1* plays important roles in embryo implantation by inhibiting lipid peroxidation (Jia et al., 2016). Furthermore, a role for ApoA1 in early embryonic development has also been suggested, as this protein is produced by human preimplantation embryos, and increased *ApoA1* levels are present in spent culture media containing blastocysts of high morphologic grade (Mains et al., 2011). Sensor systems for cellular metabolism and energy are essential during early embryonic development (McBride et al., 2009). The protein encoded by the *PRKAA*1 gene is the catalytic subunit of AMPK, which is a cellular energy metabolism sensor conserved in all mammalian cells. The metabolic sensor *PRKAA1* seems to be important for embryonic development in sustaining cell polarity and advancing the cell cycle (Lee et al., 2007; Jansen et al., 2009). Finally, another upregulated gene that plays an important role in embryonic growth and development is *RICTOR*, a protein-encoding gene essential for the development of both embryonic and extraembryonic tissues, as its deficiency causes embryonic lethality in mice (Shiota et al., 2006).

## Final comments

Achieving a successful pregnancy is the main goal of all reproductive biotechnologies. Boar SP has been demonstrated to be a determinant factor for basic sperm function. Moreover, boar SP acts as a protective factor for spermatozoa in its transit through the uterus and, more importantly, as a factor that prepares the uterine environment to receive the embryo, promoting its proper development and implantation.

Therefore, broadening the knowledge of the molecular mechanisms by which SP exerts its effects at the sperm, embryo and uterine levels is prerequisite for optimal design of adequate protocols that allow improved fertility results for procedures such as AI, cryopreservation and/or embryo transfer.

Currently, due to the use of ´omics techniques, the identification of reliable biomarkers to determine sperm function and fertility seems to be more likely in the near future. The economic impact of an increase in farrowing rates but particularly in live litter size that could result from selecting boars according to the expression of certain proteins, would have a great impact on pig productivity. Similarly, these potential biomarkers could be used as tools for improving current sperm preservation procedures. Future studies based on the recent and continuous advances in ´omics techniques will contribute to the identification of reliable biomarkers that can be used as fertility indicators and additives to improve the yields derived from the application of different assisted reproductive technologies in pigs.

Regarding embryo technologies, the possibilities of an increase in the productive and reproductive parameters derived from ET procedures, by applying strategies based in molecular indicators, would hugely contribute to its broad implementation. This would, in fact, accelerate genetic improvement and improve animal welfare. A greater knowledge of the impact of the different components of SP on the creation of a favorable uterine environment for pregnancy establishment is critical to improve the performance of embryonic technologies in pigs. In this review, we showed that SP infusions during estrus affected the transcriptional expression profile of the endometrium and preimplantation embryos during early pregnancy, by positively influencing the expression of genes and pathways associated with embryonic early development and facilitating achievement of the state of tolerance by the maternal immune system. Obviously, potential effects of SP infusions on the outcomes of ET programs need to be further explored.
